# Promote or Demote? Investigating the Impacts of China’s Transferable Development Rights Program on Farmers’ Income: A Case Study from Chongqing

**DOI:** 10.3390/ijerph192113751

**Published:** 2022-10-22

**Authors:** Long Cheng, Zhengchun Xu, Jintao Li

**Affiliations:** 1 School of Political Science and Public Administration, Shandong University, Qingdao 266237, China; 2 Centre for Quality of Life and Public Policy Research, Shandong University, Qingdao 266237, China; 3 China Construction Third Engineering Bureau Group Co., Ltd., Wuhan 430070, China

**Keywords:** synthetic control method, transferable development rights, land ticket, farmer’s income, urbanization

## Abstract

China has been undergoing rapid urbanization and industrialization process since the adoption of economic reform and open-door policy in 1978, which is leading to tremendous urban growth and encroachment on rural farmland. To address the conflicts between urban development and farmland protection, the Chinese government introduced the transferable development rights (TDR) program named the Link Policy, and it was popularized nationwide given the tremendous land revenue from policy implementation. However, as farmers are the key stakeholders, the impacts of the policy on farmers’ income need to be examined and justified. Thus, this paper aims to fill this gap by taking Chongqing as a case study. The synthetic control method was introduced to construct a synthetic Chongqing without the policy implementation using the 49 municipal cities and Chongqing during 2000–2017. Findings from the analysis indicate that Chongqing’s TDR program significantly promoted farmers’ income at the beginning of the policy implementation, while the positive impacts became weak afterward. Based on these findings, a few policy suggestions including a fair revenue distribution scheme and protection of farmers’ land use rights are offered to facilitate the policy implementation and increase farmers’ household income in the coming future.

## 1. Introduction

Globally, urbanization has become an inevitable trend showing almost exponential growth since the end of the 19th century [1]. Consequently, massive migrations from rural to urban areas have significantly reshaped the landscape of urban and rural areas [2,3]. Specifically, developing countries account for a larger proportion of the world urbanization process and are facing more critical related challenges such as food security, urban poverty and inequalities in wealth and resources [4]. As the *World Cities Report 2020* indicates, more than 50% of the world’s population lived in urban areas in 2008, and the urban population will keep growing in the next few decades [5]. In the middle of the 21st century, 86% of the developed world and 64% of the developing world will be urbanized; 90% of the increase will take place in Asia and Africa [5,6], and China, India and Nigeria are projected to be the major contributors to the urban population [6,7].

After the economic reform and openness, the Chinese government has treated urbanization as an effective policy instrument sustaining its prosperous economic growth [8]. Given the existing strict household registration (hukou) system and dual track system of urban–rural land administration, China’s urbanization strategy is quite different from that of other developed and developing countries [9]. Its uniqueness can be shown as dual-track urbanization, consisting of both bottom-up and top-down paths [10,11,12]. Specifically, a few key metropolises can benefit from the prioritized policies and financial support from the central government and thus experience an accelerated urbanization process [10,11]. Concurrently, “bottom-up urbanization” occurs in the process of urbanization in many small towns and villages [13,14,15]. Given that, prefecture-level and county-level cities sprouted out dramatically from only 193 in 1978 to 672 in 2019, which resulted in the urban built-up areas expanding continuously by 1379 km^2^ per year on average from 7438 km^2^ in 1981 to 58,455.7 km^2^ in 2018 [16].

Undeniably, urbanization plays a fundamental role in boosting China’s economic growth [17,18,19]. However, related issues also appeared constantly, including rampant agricultural land acquisition, low-efficiency use of urban construction land and violation of farmers’ land use rights and interests [20,21,22,23]. Additionally, due to the massive outflow of rural migrants from rural areas to cities, farmers’ original dwellings and contracted farmland become vacant and idle, leading to the low-efficiency use of land and poor rural living conditions [24,25]. To address the issue of farmland protection and prevent rampant urban expansion, the central government introduced the Chinese version of the transferable development rights program termed the Link Policy in 2004 [25,26]. The full name of the Link Policy is “Linking the increase in urban construction land with the decrease in rural construction land”. It calls for rural resettlement and land consolidation to provide extra space for urban development. Farmers who previously lived in poor living conditions will be relocated into high-rise and centralized modern communities, and then their original homestead will be converted into farmland. In this process, extra land quotas can be generated and traded with urban developers for urban construction [27,28]. A few trials of the Link Policy showed its positive effects on resolving the urban and rural development conflicts, particularly in reaping land revenue from rural land consolidation. Thus, with the official regulations proposed in 2008, the Link Policy was soon popularized throughout the whole country [29,30].

Since the adoption of the Link Policy, local governments have shown great passion for implementing the policy and also formed a few successful localized practices, such as the Land Ticket Program in Chongqing [31,32], Demolish and Combine Courtyards in Chengdu [33], Homestead-for-Apartment in Tianjin [34] and the Flat-for-Flat Scheme in Zhejiang Province [35]. The Link Policy has been regarded as an important method to resolve the conflicts of urban development and farmland preservation in China’s rapid urbanization process. As an institutional innovation, the Link Policy has developed an informal rural land market accelerating the interaction of urban and rural land elements [36]. Additionally, the transactions of land quotas created from the Link Policy implementation accumulated great land revenue for rural development, offering upgraded living conditions in rural areas and also changing farmers’ lifestyles significantly [37].

A review of the existing related literature shows that previous researchers focused more on the introduction and description of the Link Policy, including the local practices and the pros and cons of the policy. For example, Li et al. [37] introduced the implementation of the Link Policy in Jilin province and explained that the policy can facilitate rural land use optimization. Gao et al. [38] revealed the rural settlement paths under the Link Policy by using cases of Chongqing, Chengdu and Shanghai. Chen et al. [39] introduced Chongqing’s mode of implementing the Link Policy termed “Dipiao” or land tickets. Cheng, Liu, Brown and Searle [30] explored farmers’ satisfaction with the implementation of the Link Policy and analyzed the underlying factors. Long, Li, Liu, Woods and Zou [25] investigated the impacts of the Link Policy on the phenomenon in the process of urbanization termed “village-hollowing”. Huang et al. [40] analyzed the controversial commentaries of the Link Policy and offered relevant suggestions for future improvement. Liu et al. [41] discussed the impacts of the withdrawal of homesteads under the Link Policy in Chengdu, Sichuan Province. Huang et al. [42] applied a policy process analysis method to reveal the drivers of the formation and adoption of the Link Policy in China.

Previous studies on the Link Policy offer a solid foundation for understanding the Link Policy. However, the studies on the impacts of the Link Policy, particularly those on farmers’ income, are very limited. Thus, this paper aims to investigate the policy impacts on farmers’ income by using the synthetic control method and analyzing data collected on a regional scale. The remainder of the paper is arranged as follows: Section 2 offers a comprehensive introduction of the Link Policy, including the policy background and design. Section 3 presents the sources of data and introduces the key methodology used in this paper. Section 4 presents the data analysis process revealing the impacts of the Link Policy on farmers’ income, followed by further discussion and the conclusion of this research in Section 5 and Section 6.

## 2. Understanding the Chinese Version of the TDR Program: The Link Policy

### 2.1. The Proposal of the Link Policy

China has been undergoing tremendous urbanization since the launch of its economic reform and openness in 1978. In this process, two fundamental reforms had far-reaching impacts on China’s development and urbanization: the land administration reform in 1988 and the fiscal reform in 1994 [43]. The land administration reform separated land use right from land ownership and enabled the trading of land use rights. The fiscal reform came under the situation that the central government faced financial difficulties and therefore changed the share of financial income between the local government and central government. A larger proportion of tax revenue was taken by the central government while the local government just kept a few types of taxes. However, the local government was still responsible for its own development, and the revenue from the transaction of land use rights remained with the local government. Thus, local authorities shifted their focus to granting land use rights to developers so as to achieve more financial income [44]. This generated the famous land finance in China which sustained and boosted China’s economic miracle and development. However, the chasing of land finance resulted in a few critical issues, among which the issue of conflicts between farmland preservation and urban development was the most severe and tough one for the central government [45,46].

Aiming to address this issue, the Chinese government introduced a few policies. For instance, China created the land quota system under the land use master plan scheme. Under this system, the quota for construction land during the planning period is strictly allocated each year from the central government to the local government hierarchically, which indicates that the annual quota for urban construction use has been strictly restrained and pre-set in the planning process [47,48]. However, given the rapid economic growth and urbanization process, urban areas, particularly in the metropolises, are facing a lack of space for development [49]. Concurrently, due to the massive outflow of rural migrants to urban areas, rural China is becoming vacant and declining with low-efficiency use of land and a lack of working forces, which enlarged the urban–rural discrepancies. Given that, the Chinese government turned to the coordination of urban–rural development by introducing the transferable development rights (TDR) program. The Chinese version of TDR is termed the Link Policy, which enables offering more spaces for urban development without breaking through the current quota system [50]. More importantly, the Link Policy can bridge the gaps between urban and rural areas with the transfer of development rights and further achieve the goal of poverty alleviation and rural revitalization.

By reviewing the policy documents, the prototype of the Link Policy can be traced back to the *Decision on Deepening Reform and Strict Land Management* in 2004, which calls for the implementation of the “Building a New Socialist Countryside” strategy (proposed in China’s “11th Five-Year Plan”) from the perspective of land administration system so as to achieve the sustainable development of economy and society [29,51]. Specifically, it indicated “encouraging rural construction land consolidation” and requires that “the increase in urban construction land should be linked to the decrease in rural construction land”. Under the premise of keeping the total amount of construction land and farmland intact, vacant rural homesteads could be consolidated into farmland, and then the generated land quota could be transferred for urban development [52]. In October 2005, the Link Policy was approved by the Ministry of Land and Resources to be firstly implemented in a few trial areas, including Shandong, Sichuan, Jiangsu, Hubei, Guangdong and Tianjin. Reflecting on the experiences from the pilot implementations, the official policy document entitled *Regulations on Implementing the Link Policy in Pilot Areas (No. 138, 2008)* was enacted, and the Link Policy was implemented nationwide thereafter.

**Figure 1:**
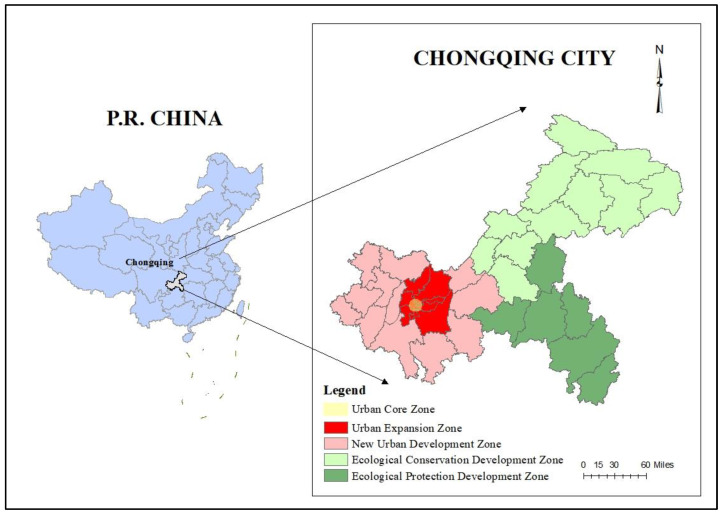
Chongqing administrative region.

Among these trials of the Link Policy, the model of Chongqing has been regarded as a successful policy innovation from the supply side of land resources. As shown in Figure 1, Chongqing is the only municipality in inland China and serves as the economic center of the upper Yangtze River area, which has an area of 82.4 thousand km^2^ and a population of 32.12 million (as of 2021). It has five major functional zones: Core Urban Zone, Expansion Urban Zone, New Urban Development Zone, Ecological Conservation Development Zone and Ecological Protection Development Zone. However, significant disparities do exist among regions, including urban and rural natural conditions, resource endowments, development status and development potentials.

Aiming to address the urban–rural development disparities, Chongqing was selected as one of two pilot areas to implement the Comprehensive Reform for Urban–Rural Integrated Development in 2007. Following this strategy, the implementation of the Link Policy has been taken as an effective policy instrument to coordinate urban and rural development. Different from the traditional practice, the policy implementation in Chongqing was termed the Land Ticket Transaction. “Land ticket” is a metaphorical expression of the tradable land use quota generated by rural construction land consolidation, and these quotas can be traded, crossing the boundary of the township with the approval of the central government [30,31,53]. The trading platform, called the Chongqing Rural Land Transaction Centre (CRLTC), was established in 2008 and was in charge of responsibilities including the registration and administration of property rights; transactions of rural property rights; distribution of land quota revenues; and offering consultations for property rights selling, bidding and auction.

### 2.2. The Link Policy vs. Transferable Development Rights

Given that the Link Policy has a few advantages, particularly in resolving land use conflicts between urban and rural areas and creating great land revenue for local governments, it has been widely welcomed and popularized nationwide. Generally, the design of the Link Policy can be regarded as a hybrid policy of compulsory land acquisition, displacement and resettlement and transferable development rights [54]. Specifically, with the implementation of the Link Policy, farmers with poor but spacious living conditions will be relocated to centralized settlements while their original homestead will be consolidated into farmland. At this stage, extra land quotas can be created and will be transferred to urban developers through transactions. Payment by developers for using these quotas will go to rural areas for the construction of new dwellings and poverty alleviation. Urban developers can apply the land quotas at the urban fringe for more construction use with land acquisition under the local authorities [55]. The transfer of land quota from rural to urban areas can be regarded as the transfer of development right in TDR, although development right is currently an informal right in China’s land administration system [56,57]. Therefore, the Link Policy has been called the Chinese version of TDR by many researchers [44,58].

TDR is a market-oriented planning instrument in the US and has been implemented for more than sixty years [59]. TDR first appeared in the 1860s, when many local authorities in the US faced challenges in resolving constraints the zoning system laid on urban development. To be more specific, under the TDR program, development rights can be transferred from sending areas that should be protected to receiving areas that need extra and more intensive development. Users of development rights in the receiving areas should pay the owners of sending areas for protecting the status quo of land use, and basically, eminent domain will be implemented [60,61]. As described, the implementation of the Link Policy is similar to that of the US TDR to some extent, given that more development space is offered to urban areas while the development of rural areas is restricted. The sending areas in TDR can be seen as the rural settlements in the Link Policy, while the receiving areas correspond to the more developed urban areas.

However, differences do exist between the Link Policy and TDR program given the different land administration systems in China and the US. Cheng, Brown, Liu and Searle [37] indicate that there are three main differences: The first is the difference in land ownership. Rural land in China is owned collectively instead of by farmers themselves. Farmers only possess land use rights, and thus they cannot directly negotiate with the developers for compensation like their counterparts in the US. The second is the different actions in the sending areas. In the Link Policy, the sending areas of development rights are the rural settlements which will be converted into farmland, while those in the US TDR should remain unchanged. The third is the different actions in the receiving areas. In the Link Policy, urban developers will basically apply the land quotas (development rights) to the urban fringe, causing urban expansion, while the US TDR calls for more intensive use of land.

### 2.3. The Link Policy in Chongqing: Land Ticket Program

**Figure 2:**
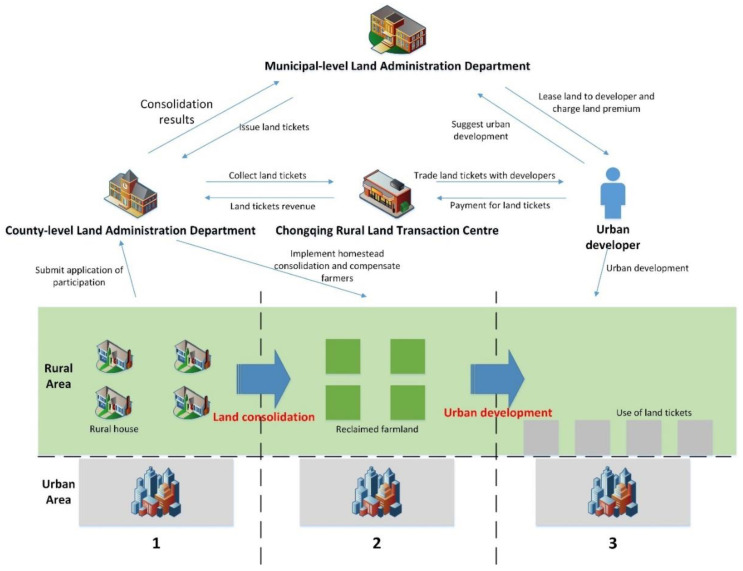
Land Ticket Program transaction process.

The Land Ticket Program is a localized practice of the Link Policy proposed by Chongqing Municipality. Following the general regulations of the Link Policy, the Land Ticket Program calls for the protection of farmland through rural land consolidation and thus offers a greater land quota for urban construction [30]. The innovation and breakthrough side is that the transaction of land quotas can be within the whole city instead of at the county level as general regulation requires [62]. Moreover, market-oriented institutions appear in the implementation process, in which the establishment of the Chongqing Rural Land Transaction Centre offers a platform for quota transactions and facilitates the implementation process. The implementation of the Land Ticket Program can be summarized in four stages as follows:

The first is the consolidation of the rural settlements. Farmers in selected areas can apply for participation in the Land Ticket Program at their will. The rural committee will review the application and initiate the consolidation process once the rural household meets the basic requirements such as idle or vacant homesteads. Once the homestead consolidation has been finished and passed the review by local officials, land tickets will be issued to rural households, labelling the acreage of the consolidated homestead. The second is the transaction of the land tickets. Rural households can pool their land tickets at the Chongqing Rural Land Transaction Centre, where urban developers can buy these tickets through bidding, listing and auction. Once the transaction is finished, developers need to pay for the use of land quota, and this part will go to rural households and collectives with a ratio of 85 to 15. Additionally, Chongqing also proposed a price protection scheme to ensure that farmers’ minimum revenue for land ticket transaction is no less than 120,000 CNY/mu and that the minimum revenue for rural collectives is no less than 21,000 CNY/mu. The third is the application of land tickets for urban developments. Land tickets have been the premise for developers who want to bid for urban construction land at the land market. If the developers succeed in the land market, their payment for previous land tickets can be used to offset parts of the land conveyance fee. However, if they fail, the payments for land tickets will be returned. The more detailed implementation process of the Land Ticket Program is shown in Figure 2.

**Figure 3:**
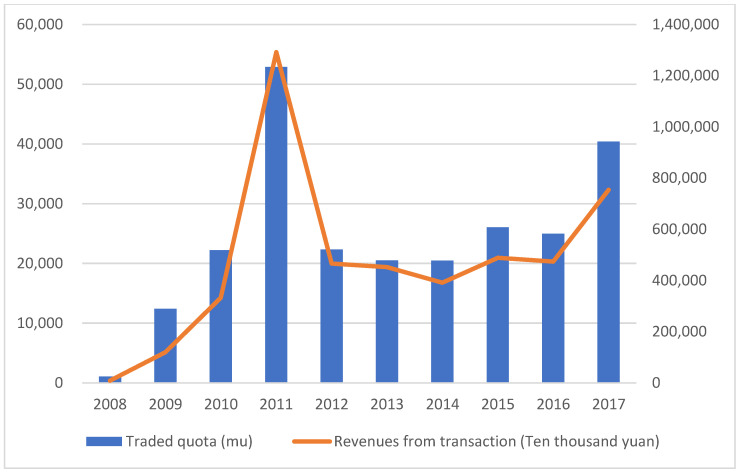
Trend of land ticket transactions from 2008 to 2017.

Since its adoption, Land Ticket Program has been widely reported as a successful instrument for coordinating urban and rural development with market approaches. It created tremendous land revenue and boosted the local economy. As of 2017, more than 0.24 million mu land tickets have been created and traded, with more than CNY 47.8 billion in land revenue [63]. The trend of land ticket transactions from 2008 to 2017 is shown in Figure 3.

## 3. Methodology

### 3.1. Data Collection and Description

Following the calculation steps of SCM, Chongqing was selected as the treatment group which implemented the land tickets since 2008 in this study, while 49 prefectures in Hunan, Hubei, Guizhou and Sichuan that have not implemented the program were selected as the control group given that they have similarities in development conditions and geographical locations to Chongqing. Following the previous research on farmers’ income and the principles of SCM, farmers’ income was selected as the dependent variable while rural mechanization level, farmers’ average farmland, average financial expenditure, per capita highway mileage and electricity consumption were used as independent variables [64,65]. All these panel data are from statistical yearbooks from the national to regional and province levels during 2000–2017. Policy documents related to the Land Ticket Program are all from Chongqing Rural Land Transaction Centre. For more detailed descriptions of all variables, please refer to Table 1. Two time frames were set, before and after the Land Ticket Program was implemented in Chongqing (2008): (1) 2000–2007 and (2) 2008–2017.

### 3.2. Synthetic Control Method

Aiming to investigate the impacts of the transferable development rights on farmers’ income, the synthetic control method (SCM) was applied in this research. SCM is a statistical method for the evaluation of the treatment effect during comparative studies; it was first developed by Abadie et al. [65] and further extended by Abadie et al. [63]. SCM has several advantages over alternative approaches to evaluating policy impacts. First, it offers an approach suitable for when there is a small number of treated units and control units, which is often the case when population-level health interventions are being evaluated. Second, unlike DiD approaches, SCM does not rely on parallel preimplementation trends. Given that it is sometimes difficult to establish whether the parallel trend assumption is met, this method provides a useful supplementary method to be used with DiD. Finally, SCM allows for unmeasured time-varying confounders, whereas DiD only allows for measured time-varying confounders. Therefore, SCM has been treated as an effective approach to evaluating the policy impacts and is widely used in social sciences. The rationale of SCM in this paper is to find a weighted combination of control prefectures to formulate a “fake” or synthetic Chongqing where the Land Ticket Program has never been implemented. The policy impacts on farmers’ income can be identified by analyzing the differences between the development pathways of the real Chongqing and the synthetic one. Compared with the difference-in-difference (DiD) method, the first and foremost advantage of SCM is that it can mitigate heterogeneity among different regions and make the real Chongqing and synthetic Chongqing comparable.

Regarding the SCM calculation, a more detailed operation process can be found in Abadie’s works [64,65]. Here, SCM in this paper is briefly introduced as follows:

It is assumed the collected data are from *P* + 1 areas and farmers’ income during the period of *T*, in which the Land Ticket Program was implemented in area *i* at $T_{0}$, while the rest of the areas selected were not affected by the program. $Y_{\mathrm{it}}^{1}$ refers to farmers’ income in the policy-implemented area at time *t*, while $Y_{\mathrm{it}}^{0}$ indicates the income from farmers not affected by the program. $\alpha_{\mathrm{it}}=Y_{\mathrm{it}}^{1}-Y_{it}^{0}$, which indicates the policy impacts on farmers’ income. Variable $D_{\mathrm{it}}$ is the dummy variable indicating whether the policy has been implemented. If the policy was implemented in area *i*, the value of $D_{\mathrm{it}}$ is 1, otherwise the value is 0. Thus, farmers’ income in area *i* at time t can be measured as follows:

(1) $$Y_{\mathrm{it}}=Y_{\mathrm{it}}^{1}+\alpha_{\mathrm{it}}D_{\mathrm{it}}$$

When *t >* $T_{0}$, $\alpha_{\mathrm{it}}=Y_{\mathrm{it}}^{1}-Y_{\mathrm{it}}^{0}=Y_{\mathrm{it}}-Y_{\mathrm{it}}^{0}$. Given that the value of $Y_{\mathrm{it}}^{1}$ is observable, the value of $\alpha_{\mathrm{it}}$ can be measured by $Y_{\mathrm{it}}^{0}$, which is calculated below.

(2) $$Y_{\mathrm{it}}^{0}=\delta_{t}+\theta_{t}^{'}Z_{i}+\lambda_{t}^{'}{\mu \;}_{i}+\varepsilon_{\mathrm{it}}$$

where $\delta_{t}$ is the fixed effect of time, indicating farmers’ income at different times. $Z_{i}$ is a vector with N dimensions, indicating the observable covariances that are not affected by the policy and time. $\theta_{t}^{'}$ is an unknown parameter vector with N dimensions, and $\lambda_{t}^{'}$ is an unobservable common factor with F dimensions; $\mu_{i}$ refers to the fixed effects which are not observable, while $\varepsilon_{\mathrm{it}}$ demonstrates the unobservable intermediate strike in area *i* with an average value of 0.

Assuming that *W* is a weighted vector with *K* dimensions, saying $W=\left(w_{1}^{*},\ldots {,w}_{i-1,}w_{i+1},\ldots {,w}_{k+1}\right)$, and setting $w_{k}\in w$, it follows that $w_{k}>0$ and $\sum_{k}w_{k}=1$. All the vector w which meets the aforementioned conditions represents a synthetic control group, and the results can be measured as the weighted average of all control groups as shown in Formula (3).

(3) $$\sum_{k\neq i}W_{k}Y_{\mathrm{kt}}=\delta_{t}+\theta_{t}^{'}\sum_{k\neq i}W_{k}Z_{k}+\lambda_{t}^{'}\sum_{k\neq i}W_{k}{\mu \;}_{k}+\sum_{k\neq i}W_{k}\varepsilon_{\mathrm{kt}}$$

where *k* represents the areas in which the Land Ticket Program was not implemented.

If the optimally weighted matrix exists as $W=w_{1}^{*},\ldots ,w_{i-1}^{*},w_{i-1}^{*},\ldots ,w_{k+1}^{*}$, then Formula (4) is enabled:

(4) $$\sum_{k\neq i}W_{k}^{*}Y_{k1}=Y_{k1},\ldots ,\;\sum_{k\neq i}W_{k}^{*}Y_{{\mathrm{kT}}_{0}}=Y_{{\mathrm{iT}}_{0}}\;\mathrm{and}\;\sum_{k\neq i}W_{k}^{*}Z_{K}=Z_{i}$$

Abadie, Diamond and Hainmueller [64] proved that if $\sum_{k\neq i}^{T_{0}}\lambda_{t}^{'}\lambda_{t}$ is a nonsingular matrix, then

(5) $$Y_{\mathrm{it}}^{0}-\sum_{k\neq i}w_{k}^{*}Y_{\mathrm{kt}}=\lambda_{t}^{'}(\mu_{\mathrm{kt}}-\sum_{k\neq i}w_{k}\mu_{k})+\sum_{k\neq i}w_{k}^{*}\varepsilon_{\mathrm{it}}-\varepsilon_{\mathrm{kt}}$$

In the equation above, if the size of all units is big enough prior to the policy implementation, the right-hand side of the formula approaches 0. Thus, $\sum_{k\neq i}w_{k}^{*}Y_{\mathrm{kt}}$ can estimate $Y_{\mathrm{it}}^{0}$ very well. The unbiased estimator of policy impacts can be described as

(6) $$\overset{\wedge }{\alpha_{\mathrm{it}}}=Y_{\mathrm{it}}-\sum_{k\neq i}w_{k}^{*}Y_{kt},t\in \{T_{0}+1,\ldots ,T\}$$

## 4. Results

### 4.1. Modeling Results

As of 2017, all districts and counties in Chongqing have participated in the Land Ticket Program. By comparing the economic conditions of Chongqing with four other selected provinces from 2000 to 2017 (see Table 2), farmers’ income and rural mechanization level in Chongqing are significantly lower than the data from the control group, while average financial expenditure, per capita highway mileage and per capita electricity consumption in Chongqing are significantly higher than those in four other provinces. There is no difference in the farmers’ average farmland. Concurrently, by comparing the economic conditions during the periods of 2000–2007 and 2008–2017, it can be seen that they all have significant differences with the overall period of time from 2000 to 2017. Given that, the following part will try to examine whether the Land Ticket Program had positive impacts on local economic development.

The SCM was run in STATA using the code package of Synth developed by Abadie, which contributes to analyzing the impacts of the Land Ticket Program on farmers’ income. Table 3 presents the comparison between the composite value and the true value of the predictor variables in Chongqing, which indicates that the model fit is very good. In particular, farmers’ average farmland and fiscal expenditure in two groups are almost the same.

Table 4 shows the weight of each unit in the control group which forms the synthesized Chongqing, in which eight prefectures match the target Chongqing, including Chengdu (0.102), Ziyang (0.317), Huangshi (0.022), Shiyan (0.285), Yichang (0.037), Huangang (0.189) and Liupanshui (0.049).

**Figure 4:**
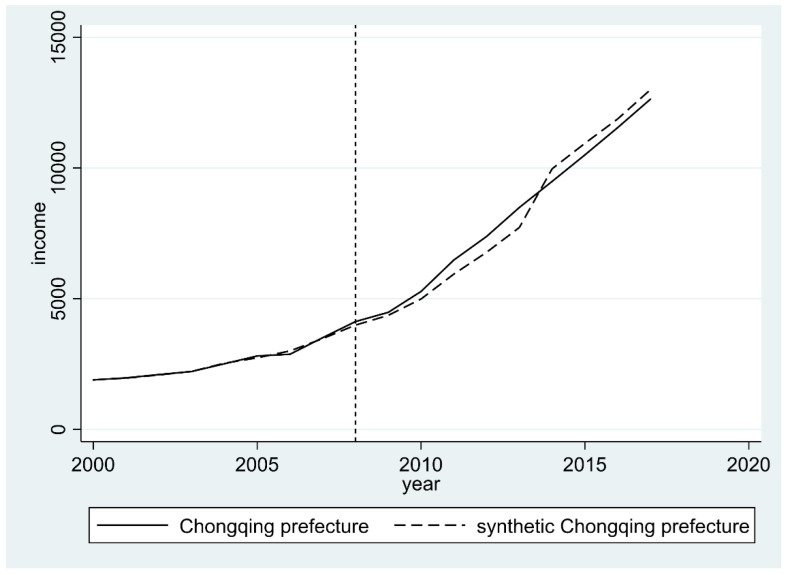
Changes in farmers’ income in real Chongqing and synthetic Chongqing.

Figure 4 indicates the changes in farmers’ income in Chongqing and synthetic Chongqing from 2000 to 2017, in which the vertical dotted line represents the starting year of the Land Ticket Program, i.e., 2008. As shown, on the left-hand side of the 2008 line, the growth path of farmers’ income in real Chongqing is almost the same as that in synthetic Chongqing, indicating that data used in SCM meet the requirements and the synthesized Chongqing can well represent the real Chongqing from the perspective of farmers’ income. On the right-hand side of the line 2008 (after the policy was implemented), some differences appear between the treatment group and the synthetic group. To be more specific, during the years 2008 to 2014, farmers’ income in real Chongqing is higher than that in synthetic Chongqing, while the pace of increase in farmers’ income in synthetic Chongqing is slowing down, resulting in the farmer’s income in real Chongqing being significantly lower than that in synthetic Chongqing.

**Figure 5:**
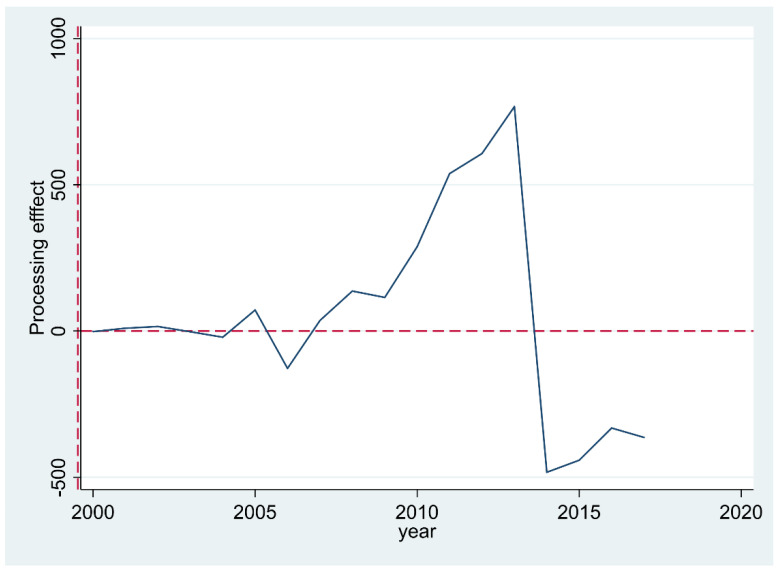
Treatment effect of the Land Ticket Program.

To view the treatment effect more clearly, STATA was used to produce Figure 5, which shows the treatment effect of the Land Ticket Program. The results show that the early stage of the policy implementation (2008–2014) can be seen as a positive treatment effect, while the treatment effect in the latter part of the policy implementation (2014–2017) becomes negative.

### 4.2. Robustness Test and Placebo Test

Given that the significance of the non-parametric results from SCM from economic and statistical perspectives cannot be justified through large sampling statistics, a robustness test is needed to ensure the reliability of the analysis. Referring to previous studies [66], if the value from the synthetic group matches with the real group, a robustness test is of practical significance. As analyzed in Section 4.1, the SCM results meet the requirements for the robustness test. Thus, the robustness test was implemented in the following process:

It is necessary to justify the differences in the predicted variable so as to examine the effectiveness of the modeling results. The robustness test in this research was implemented with the approaches that Abadie, Diamond and Hainmueller [64] proposed. The key rationale is as follows: Assuming all the units in the treatment group implemented the Land Ticket Program in 2008, synthetic control analysis is needed for each unit in the control group. Comparing the policy treatment effects of Chongqing and other assumed prefectures that implemented the Land Ticket Program, if the policy effect in Chongqing is significantly different from other prefectures in the control group, it is believed that the Land Ticket Program in Chongqing has significant positive impacts on farmers’ income.

**Figure 6:**
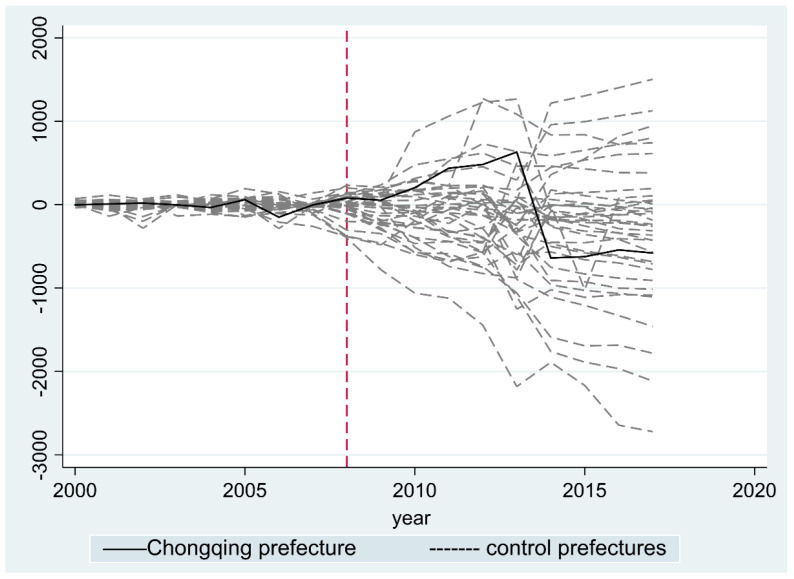
Robustness test result.

Additionally, the robustness test was supplemented with a placebo test to make the modeling results more reliable. Prior to that, prefectures with larger root mean square percentage error (RMSPE) values in the SCM were removed given that a larger RMSPE indicates the bad imitative effect of the synthetic group before the policy was implemented. The removed prefectures are places with a value of RMSPE over 1.5 times that of Chongqing, including Ziyang, Jingmen, Changsha, Xiaogan, Xiangxi, Bijie Guangyuan, Neijiang, Shiyan, Nanchong, Guang’an, Guiyang and Liupanshui. The final robustness test result can be found in Figure 6, indicating the different distributions of the rest of the prefectures, in which the solid line indicates Chongqing and the dotted lines indicate other prefectures.

The placebo test results show that the imitative effect before the Land Ticket Program was implemented in 2008 is good enough, and the solid line shows a pattern of waves. To be more specific, the upwards period is from 2008 to 2013, when the majority of the dotted lines are below the solid line, indicating the policy impacts in real Chongqing are significant. However, during the period of 2014 to 2017, the solid line declines sharply and is below the majority of the dotted lines, indicating the policy impact is not significant at this stage.

## 5. Discussion and Policy Recommendations

This paper incorporates the SCM to analyze the impacts of the Land Ticket Program on farmers’ income. The results show that the Land Ticket Program significantly increased farmers’ income at the early stage of implementation, while the impacts became weak afterward. This is due to the great passion of participants when the policy was first introduced. Correspondingly, the bonus of the policy can be easily seen, as farmers’ income significantly increased at that time. However, with time, a side effect of the policy implementation appears to result in a negative externality. Farmers’ income in real Chongqing is even lower than that in synthetic Chongqing. Given that, the results will be discussed at different stages of implementation separately.

### 5.1. Discussion for the Promotion Period (2008–2013)

The flow of factors between urban and rural areas has optimized the allocation of factors between the regions, boosting the rapid growth of the local economy and thus the income of farming households. According to the design mechanism of the land ticket trading system, the establishment of the platform of Chongqing Rural Land Exchange at the early stage of the land ticket trading system unblocked the spatial flow between urban and rural areas in the form of “land tickets”, breaking the long-standing barriers of the urban–rural dualistic system and mechanism, thus enabling inter-regional factors to move freely within the city. This has enabled the free movement of inter-regional factors within the city. At the same time, under the price mechanism of the market economy, each market player takes the initiative to seek its own needs, and in order to obtain construction land indicators to meet local development, the region, with sufficient capital elements of its own, exchanges capital elements for relatively scarce construction land indicators in order to better achieve regional economic and industrial development. The areas that provide surplus construction land indicators will also receive the capital elements needed for economic development, which will solve the development problems caused by the shortage of capital elements in the area while also providing farmers, collective economic organizations and local governments with part of the proceeds.

The increased enthusiasm of both buyers and sellers to participate and the increased activity of the market has led to an increase in the property income of farm households. On the one hand, the local government’s vigorous promotion and support of the land ticket trading system is a favorable driver for increasing farmers’ income. As the defender of public interests, the government has a certain degree of authority and reliability. At the initial stage of land-bill trading, legal entities involved in land-bill trading are allowed to enjoy part of the policy dividends, which increases the enthusiasm of market players for participating in land-bill trading and enhances the confidence of each legal entity in achieving profitability after purchasing land-bills. On the other hand, the quality and quantity of residential land and construction land available for conversion into land ticket indicators at the initial stage of land ticket trading is substantial, and the land ticket is a favorable policy for the visualization of idle and abandoned construction land assets in rural areas. In the end, farmers will also receive a corresponding property income.

### 5.2. Discussion for the Demotion Period (2014–2017)

This paper argues that the growth in farm household income between 2014 and 2017 cannot be identified as being influenced by the Land Ticket Program rather than other factors. Therefore, the effect of the Land Ticket Program on the growth of farm households’ per capita disposable income from 2014 to 2017 is not significant.

The distribution of income under the Land Ticket Program is unreasonable and unfair, which undermines the rights and interests of farming households. On the one hand, the government, as an agent, acts as the sole agent for the land tickets provided by farmers in the transaction process, and because of the scattered and large number of individual farmer groups, individual farmers do not have the opportunity to participate in direct negotiations with buyers. On the other hand, the government’s dual role as the developer of the land ticket transaction and revenue distribution system and the direct beneficiary of the benefits is such that the government receives the corresponding land premium when the transaction is concluded between the buyer and the seller, and thus the government’s demand for raising the transaction price of the land ticket is not obvious. As long as the purchaser offers more than the stipulated benchmark price, the government will sell the corresponding land premium. For example, on 11 May 2009, the transaction price of the land ticket was only CNY 0.2 million per mu higher than the benchmark price. As a result, the information asymmetry between farmers and the government has directly led to a reduction in farmers’ income from land stamps and a certain degree of damage to their rights and interests.

The land ticket trading system is not sufficiently robust, and the benefits received by regions and farmers are difficult to sustain in the long term. On the one hand, although the land transaction can enable the less developed regions to obtain a certain amount of short-term capital gains through the sale of construction land indicators, these regions still need sufficient construction land indicators in order to achieve long-term development. Even if these less economically developed regions could purchase the required land development rights through the capital element, the cost would be much higher than the proceeds from the sale of land development rights in the early stages, which means that the economic benefits from the sale of the land stamps in the early stages would not be sufficient to meet the needs of long-term development. In this respect, the land transaction system is not conducive to the long-term development of local industries. On the other hand, there is limited space for land development, and the total amount of land in rural areas that can be used for reclamation to generate land stamps is limited, and with certain restrictions on the amount of land that can be used for reclamation, the total amount of land that can be used to generate land stamps is greatly reduced. When the majority of farmers have reclaimed their homesteads and building land as arable land, they will face a situation where there will be no construction land available. As a result, farmers will not always be able to rely on the sale of land stamps in exchange for compensation income, and the lack of income sources will put farmers in a difficult position. At the same time, the cost of building a new house after the demolition of the old one is much higher than the income from the reclamation of the house, and many farmers will face the problem of spending all their savings or even taking out loans from relatives or banks, which indirectly increases the burden on farmers.

The standard of reclamation for land ticket transactions varies, and the cultivated land after reclamation is not strong enough. On the one hand, the reclamation of residential and construction land is usually carried out by the relevant construction units at a cost-binding rate, as farmers are not in a position to carry out the reclamation themselves. The cost of reclamation will be deducted from the price of the land transaction, which inevitably leads to a reduction in the farmers’ income. On the other hand, third-party organizations are profit-seeking, and the problem of inadequate cultivated land after reclamation often arises. Even though the relevant Chongqing authorities have proposed standards for reclamation, there is always the potential for inadequate cultivation and unsatisfactory farming yields as a result of inadequate reclamation techniques and unclear regulatory systems.

Therefore, we cannot conclude that it is the Land Ticket Program that has increased the disposable income of farming households rather than other factors. Therefore, this paper concludes that the implementation of the land ticket trading system had an insignificant effect on increasing the per capita disposable income of farm households during the period 2014–2017.

### 5.3. Policy Recommendations

The implementation of the Land Ticket Program has provided an important reference value for the integrated development of urban and rural areas. The Land Ticket Trading System, in the form of a “ticket”, has enabled the mutual flow of urban and rural elements, breaking down long-standing barriers to the flow of elements between urban and rural areas; breaking down institutional barriers to fair trade and the free flow of elements between urban and rural areas; and creating a virtuous circle of talent, land, capital, industry, information and other elements converging between urban and rural areas. As a major innovation in the linkage policy, the land ticket trading system has not only eased the tight supply of urban construction land but also enabled idle collective construction land in rural areas to realize its asset value. It has also improved the overall efficiency of rural agricultural land and ensured the income of rural households.

From the results of the study, it can be seen that the effect of the Land Ticket Program on the income growth of rural households was not always significant. Although the land transaction system has achieved some success as a pioneering experiment in the reform of the rural land system, the actual effect on economic development shows that the transfer of land development rights accelerates the economic development of urban areas, while the economic gains in rural areas are not significant except for a certain amount of capital gains. Therefore, this paper proposes the following policy recommendations to address the problems of the Land Ticket Program:

(1) Corresponding to the issue of unreasonable distribution of proceeds, a fair and effective system for the distribution of proceeds from land tickets should be established. Farmers will participate in the initial distribution of the land ticket transaction and the distribution of the land premium when the land ticket is granted and will adjust the proportion of land ticket revenue in a fair and reasonable manner. At the same time, the price of land development rights is introduced, and the differential land rent premiums generated in the process of the land transaction are reasonably distributed between urban and rural areas, and between the land producing and using areas in accordance with the principle of “fairness and efficiency”, with a certain proportion of the premiums being used as a development fund for the rural areas where the land tickets are produced. A certain percentage of the land rent premium will be used as a development fund for rural economic organizations in the land where the land stamps are produced, so as to feed rural infrastructure construction.
(2) Aiming to address the problem of insufficient income for farmers, a land reclamation guarantee mechanism and farmer skill training are needed. On the one hand, in conjunction with the industrial layout of the new socialist countryside, the reclaimed land should be incorporated into modern industrial construction to ensure that the reclaimed construction land can effectively create wealth for farmers in the long term, thereby increasing their income; on the other hand, the training of new skills of farmers should be strengthened to improve their livelihood skills after participation in the land ticket trading system, so as to lengthen the positive impact of the policy and to send them on their way.
(3) Recommendations to address the issue of inconsistent standards of reclamation and improve the process of land reclamation are as follows: First, strengthen the qualification audit of third-party reclamation agencies, and strictly control technical standards to enhance the grade of reclaimed arable land. Second, make effective risk assessments of the reasonableness and necessity of construction land reclamation, raise the threshold for construction land reclamation and eliminate the phenomenon of reclamation for the sake of reclamation. Third, establish a sound monitoring and review mechanism, severely punish profit-seeking behavior and do a good job of looking back.

## 6. Conclusions

This paper focuses on the Chongqing Land Ticket Program, the Chinese version of TDR, and uses the synthetic control method as the research method, selecting Chongqing as the treatment group and 49 cities and states in Hunan, Hubei, Sichuan and Guizhou as the control group and simulating a synthetic Chongqing using development data reflecting per capita disposable income of farmers in the treatment and control group areas from 2000 to 2017. The policy effects of Chongqing’s Land Ticket Program were analyzed by comparing the values of per capita disposable income of farmers between the real Chongqing and the synthetic Chongqing. The results of the study show that the Chongqing land ticket trading system has a significant and then insignificant effect on farmers’ per capita disposable income during the study period. In other words, the increase in farmers’ per capita disposable income was largely influenced by the land ticket trading system at the beginning of the land ticket trading period, while the effect of the land ticket trading system on the increase in farmers’ per capita disposable income was insignificant at the end of the land ticket trading period.

Given the data availability, this paper still has a few limitations which represent directions for work in this area: (1) The selection of variables was not perfect. In this paper, one outcome variable and five control variables were selected. Although the variables were selected with reference to previous studies and the principles of variable selection were strictly followed, it was not possible to select all the variables. Future studies should expand the range of variables selected to filter out more relevant variables. (2) The time span needs to be lengthened. In this paper, due to the limitation of the data on Chongqing land ticket transactions, the original data time period was chosen as 2000–2017. In future studies, adjustments should be made in a timely manner according to the latest data to capture the direction of changes in policy effects so that corresponding adjustments and optimizations can be made in a timely manner.

## Figures and Tables

**Table 1:** Data sources and description.

Variable	Description	Source
Farmers’ income	Total amount of money that farmers can use for consumption and savings	China Statistical Yearbook and Statistical Yearbooks of four selected provinces
Rural mechanization level	Total power of agricultural machines divided by rural population	Statistical Yearbooks of four provinces and the Provincial and Municipal Statistics Bureau
Farmers’ average farmland	The overall farmland divided by the rural population	China Statistical Yearbook and Statistical Yearbooks of four provinces
Average financial expenditure	General public expenditure divided by the total area	China Statistical Yearbook and Statistical Yearbooks of four provinces
Per capita highway mileage	Total rural road mileage divided by the rural population	China Statistical Yearbook and Statistical Yearbooks of four provinces
Per capita electricity consumption	Rural electricity consumption divided by the rural population	China Statistical Yearbook and Statistical Yearbooks of four provinces

**Table 2:** Comparison of economic development between Chongqing and the other four provinces from 2000 to 2017.

Variable	Policy Group Mean	No Policy Group Mean	Mean Difference
Panel A (2000–2017)			
Farmers’ income (CNY)	5572.087	6083.614	−511.527
Rural mechanization level (KW)	0.457	0.808	−0.351
Farmers’ average farmland (Hectares)	0.095	0.093	0.002
Average financial expenditure (Ten thousand CNY per hectare)	3.147	1.808	1.339
Per capita highway mileage (Meters)	3.259	2.548	0.711
Per capita electricity consumption (KW/h)	282.694	256.935	25.759
Panel B (2000–2007)			
Farmers’ income (CNY)	2484.825	2793.631	−308.306
Rural mechanization level (KW)	0.303	0.487	−0.184
Farmers’ average farmland (Hectares)	0.082	0.081	0.001
Average financial expenditure (Ten thousand CNY per hectare)	0.614	0.450	0.164
Per capita highway mileage (Meters)	2.041	1.580	0.461
Per capita electricity consumption (KW/h)	156.605	180.097	−23.492
Panel C (2008–2017)			
Farmers’ income (CNY)	8041.897	8715.600	−673.703
Rural mechanization level (KW)	0.580	1.060	−0.48
Farmers’ average farmland (Hectares)	0.105	0.102	0.003
Average financial expenditure (Ten thousand CNY per hectare)	5.151	2.894	2.257
Per capita highway mileage (Meters)	4.232	3.329	0.903
Per capita electricity consumption (KW/h)	383.565	319.381	64.184

Through the *t*-test, the different statistics of Panel A, Panel B and Panel C are all significant at the 1% level.

**Table 3:** The comparison of the true value and the synthetic value of the predictor variables.

Predictor Variable	Real Value	Composite Value	Difference Value
Rural mechanization level (KW)	0.303	0.304	−0.001 **
Farmers’ average farmland (Hectares)	0.082	0.082	0.000 **
Average financial expenditure (Ten thousand CNY per hectare)	0.641	0.454	0.187 **
Per capita highway mileage (Meters)	2.041	2.04	−0.03 **
Per capita electricity consumption (KW/h)	156.605	157.055	−0.45 **
Farmers’ income (CNY) (2000)	1892.44	1901.996	−9.556 **
Farmers’ income (CNY) (2003)	2214.55	2217.611	−3.061 **
Farmers’ income (CNY) (2007)	3509.29	3473.323	35.677 **

Using the synthetic control method, the weight of each synthetic control area was calculated. ** *p* < 0.01 (two-tailed).

**Table 4:** Synthetic area weight value combination table.

Province	Variable	Farmers’ Income (CNY)	Province	Variable	Farmers’ Income (CNY)
	Area			Area	
Hunan (14)	Changsha	0		Yibin	0
	Zhuzhou	0		Guang’an	0
	Xiangtan	0		Dazhou	0
	Hengyang	0		Ya’an	0
	Shaoyang	0		Bazhong	0
	Yueyang	0		Ziyang	0.317
	Changde	0	Hubei (12)	Wuhan	0
	Zhangjiajie	0		Huangshi	0.022
	Yiyang	0		Shiyan	0.285
	Chenzhou	0		Yichang	0.037
	Yongzhou	0		Xiangyang	0
	Huaihua	0		Ezhou	0
	Loudi	0		Jingmen	0
	Xiangxi	0		Xiaogan	0
Sichuan (18)	Chengdu	0.102		Jingzhou	0
	Zigong	0		Huanggang	0.189
	Panzhihua	0		Xianning	0
	Luzhou	0		Suizhou	0
	Deyang	0	Guizhou (5)	Guiyang	0
	Mianyang	0		Zunyi	0
	Guangyuan	0		Liupanshui	0.049
	Suining	0		Anshun	0
	Neijiang	0		Bijie	0
	Leshan	0	Total	49	
	Nanchong	0	Matched prefectures	7	
	Meishan	0	Total weights	1	

## Data Availability

Data will be available on request.
